# Capturing the spatial variability of HIV epidemics in South Africa and Tanzania using routine healthcare facility data

**DOI:** 10.1186/s12942-018-0146-8

**Published:** 2018-07-11

**Authors:** Diego F. Cuadros, Benn Sartorius, Chris Hall, Adam Akullian, Till Bärnighausen, Frank Tanser

**Affiliations:** 1 Department of Geography and Geographic Information Science, University of Cincinnati, Cincinnati, OH 45221 USA; 2 Health Geography and Disease Modeling Laboratory, University of Cincinnati, Cincinnati, USA; 3 Department of Public Health Medicine, School of Nursing and Public Health, University of KwaZulu-Natal, Durban, South Africa; 4 Geographical Information Systems and Science Program, Kingston University, London, UK; 5 Institute for Disease Modeling, 3150 139th Ave SE, Bellevue, USA; 6 Africa Health Research Institute, University of KwaZulu-Natal, Durban, South Africa; 7 Heidelberg Institute for Public Health, University of Heidelberg, Heidelberg, Germany; 8 Department of Global Health and Population, Harvard T.H. Chan School of Public Health, Boston, USA; 9 School of Nursing and Public Health, University of KwaZulu-Natal, Durban, South Africa

**Keywords:** Spatial analysis, HIV prevalence, High resolution maps, Healthcare facilities, Sub-Saharan Africa

## Abstract

**Background:**

Large geographical variations in the intensity of the HIV epidemic in sub-Saharan Africa call for geographically targeted resource allocation where burdens are greatest. However, data available for mapping the geographic variability of HIV prevalence and detecting HIV ‘hotspots’ is scarce, and population-based surveillance data are not always available. Here, we evaluated the viability of using clinic-based HIV prevalence data to measure the spatial variability of HIV in South Africa and Tanzania.

**Methods:**

Population-based and clinic-based HIV data from a small HIV hyper-endemic rural community in South Africa as well as for the country of Tanzania were used to map smoothed HIV prevalence using kernel interpolation techniques. Spatial variables were included in clinic-based models using co-kriging methods to assess whether cofactors improve clinic-based spatial HIV prevalence predictions. Clinic- and population-based smoothed prevalence maps were compared using partial rank correlation coefficients and residual local indicators of spatial autocorrelation.

**Results:**

Routinely-collected clinic-based data captured most of the geographical heterogeneity described by population-based data but failed to detect some pockets of high prevalence. Analyses indicated that clinic-based data could accurately predict the spatial location of so-called HIV ‘hotspots’ in > 50% of the high HIV burden areas.

**Conclusion:**

Clinic-based data can be used to accurately map the broad spatial structure of HIV prevalence and to identify most of the areas where the burden of the infection is concentrated (HIV ‘hotspots’). Where population-based data are not available, HIV data collected from health facilities may provide a second-best option to generate valid spatial prevalence estimates for geographical targeting and resource allocation.

**Electronic supplementary material:**

The online version of this article (10.1186/s12942-018-0146-8) contains supplementary material, which is available to authorized users.

## Background

Human immunodeficiency virus (HIV) prevalence in sub-Saharan Africa (SSA) is characterized by large geographical variation [1, 2]. The overall HIV epidemic has been shown to be concentrated across clustered micro-epidemics of different geographical scales [3–6]. This evidence has been aligned with the Joint United Nations Programme on HIV/AIDS (UNAIDS) concept “know your epidemic, know your response” for the identification of geographical populations at higher risk and burden of HIV [4]. Previous studies have explored the impact of focusing resources and control interventions using a spatially-targeted allocation strategy [7–9]. Results from these studies have supported this strategy and shown that spatial targeting of interventions can substantially improve the efficiency of resource allocation, compared to a homogeneous distribution strategy [8, 10–12]. Based on this evidence, programs such as The United States President’s Emergency Plan for AIDS Relief (PEPFAR) and UNAIDS Fast-Track strategy have gradually shifted its strategy towards optimization of resource allocation including geographically relevant data [13, 14].

The implementation of spatially-targeted intervention strategies faces numerous challenges. Population-based spatial data are scarce and gathering spatial HIV data for identifying areas of high burden of the infection can be costly to implement in resource-limited settings. Some international agencies such as USAID’s Demographic and Health Survey (DHS) collect nationally representative population-based epidemiologic data from resource limited settings [1], but the surveys are not routinely conducted, and spatial data are not available for several countries where the surveys are implemented. Other surveillance systems such as the Africa Centre Demographic Information System (ACDIS), or the Centre for the AIDS Programme of Research in South Africa (CAPRISA) also include spatial information, but they are conducted in selected micro-geographical areas, limiting the generalizability of their findings to other settings or to larger geographical scales.

Alternatively, there is a wealth of clinic-based data collected from different healthcare facilities that conduct routine HIV testing and other HIV services. The feasibility of using such sources of data to explore the spatial structure of the HIV epidemic in a given setting, however, is unknown. Against this background, we address the following question: can routinely collected and readily available HIV testing data, such as those collected from healthcare facilities, be used to accurately map the broad spatial structure of the HIV epidemic? To assess whether clinic-based HIV data accurately capture the spatial structure of HIV prevalence and to identify the so-called ‘hotspots’ of infection, we conducted a series of spatial statistical analyses at two different geographical scales (national and local level), thereby offering a potentially rapid and inexpensive approach to understanding the spatial structure of HIV epidemics across differently geographic scales.

## Methods

For comparison, we conducted the analysis using data from two different geographical scales, local and national scale.

### Data sources: South Africa (local level)

Population-based local level data come from one of the most comprehensive demographic surveillance systems in Africa: the ACDIS [5, 15, 16], which is located in Hlabisa subdistrict, one of the five subdistricts in the rural district of Umkhanyakude in northern KwaZulu-Natal, South Africa (Additional file 1: Figure S1 A). This population-based surveillance system has routinely collected socio-demographic, behavioral and epidemiological information on a population of approximately 90,000 participants within a circumscribed geographic area (438 km^2^) for over a decade (Additional file 1: Figure S1 A). Along with the ACDIS is a population-based HIV surveillance and sexual behavior survey which takes place annually. We included the population-based HIV surveillance conducted in 2014 in our analysis. A total of 5174 homesteads (georeferenced to < 2 m) were included in the survey (Additional file 1: Figure S2 A). The estimated HIV prevalence using the in the population-based survey data (*pHIV*) at a sample location *i* was defined to be *pHIV*_*i*_ = *H*_*HIVi*_/*N*_*i*_, where *H*_*HIVi*_ denotes the number of sampled people from location *i* who were HIV positive and *N*_*i*_ denotes the total number of sampled individuals at location *i*.

Data sources for the clinic-based data for this study area come from the district health information system (DHIS). Antenatal clinic data from DHIS collected in 2014 from 10 healthcare facilities located in the area where the population-based surveillance is conducted were included in the analysis (Additional file 1: Figure S2 B). The antenatal healthcare facility data are collected among pregnant women attending the healthcare facilities. These data have provided invaluable information for tracking HIV prevalence and trends in most countries with generalized HIV epidemics [17]. The estimated HIV prevalence using the healthcare facility data (*cHIV*) at a healthcare facility *j* was defined to be *cHIV*_*j*_ = *f*_*HIVj*_/*N*_*j*_, where *f*_*HIVj*_ denotes the number of pregnant woman from healthcare facility *j* who were HIV positive and *N*_*j*_ denotes the total number of pregnant woman tested at healthcare facility *j*.

### Data sources: Tanzania (national level)

National level data come from the DHS conducted in Tanzania in 2011–2012 [18]. Subjects were enrolled in DHS surveys via a two-stage sampling procedure to select households. A total of 568 sampling geo-located randomly selected community clusters was included in the survey (Additional file 1: Figure S2 C). The global positioning system was used to identify and record the geographical coordinates of each DHS sample location. A total of 17,745 individuals (9756 women and 7989 men) from the selected households were eligible for the study. Further details related to the DHS methodology, study design, and data can be found elsewhere [18–20]. The method to estimate the HIV prevalence at each DHS sample location was the same as the method previously described to estimate HIV prevalence using the population-based survey (ACDIS) for the local level data. Clinic-based data from Tanzania come from antenatal clinic surveillance where HIV testing was routinely conducted to pregnant women attending these clinics in 2010 [9, 21]. Data from 132 healthcare facilities in Tanzania were included in the analysis (Additional file 1: Figure S2 D). The method to estimate the HIV prevalence at each healthcare facility was the same as the method previously described to estimate the HIV prevalence using the DHIS antenatal healthcare facility data in South Africa. Further description of data sources and maps illustrating the sampling locations are included.

### Spatial analysis

The prevalence of HIV was estimated at each sample location (for ACDIS, DHS, or healthcare facility). We used ESRI ArcGIS Desktop 10.3 [22] to generate continuous surface maps of HIV prevalence from each of the four datasets using a kriging interpolation technique, a methodology widely used in spatial mapping [23–26]. Kriging is a geostatistical method that generates an estimated continuous surface from a scattered set of points with z-values (i.e. HIV prevalence) implemented with the Geostatistics tool in ArcGIS. Kriging assumes that the distance between sample points reflects a spatial correlation that can be used to explain the observed variation in the surface. The method fits a mathematical function to the data points to determine the output value for each location. We used ordinary kriging to predict values of HIV prevalence at unmeasured locations by estimating a variogram of weighted averages of the data [27]. A secondary analysis using cokriging method was conducted to improve spatial estimations by including covariates of population density distribution, distance to the closest main road, and distance to the closest healthcare facility.

### Data sources comparisons

HIV prevalence estimations from the population-based data and clinic-based data derived from both models, kriging and cokriging, were extracted at each data point included for the ACDIS study area and Tanzania (Additional file 1: Figure S2 A, C). Partial rank correlation coefficient (PRCC) to compare population-based data and clinic-based data estimations were conducted for both ACDIS study area and Tanzania. Likewise, residuals between the two continuous surface maps of HIV prevalence generated using both types of data sources (population-based and clinic-based data) were estimated from the two different scales (local and national). A third method included the estimation of the spatial correlation between HIV prevalence calculated from the two types of data sources using bivariate local indicators of spatial autocorrelation (LISA) included in the GeoDa environment [25]. This method identifies significant spatial clustering based on the degree of linear association between HIV prevalence at a given location estimated using the population-based and the clinic-based data [26]. Maps were generated illustrating the locations with statistically significant associations along with the type of spatial association between both HIV prevalence estimations (i.e. high–high HIV, low–low, low–high, and high-low). Finally, HIV ‘hotspots’ (areas with HIV prevalence in the upper quintile estimated independently for each data source, population-based and clinic-based data [2]) were identified, then the HIV hotspots identified using both sources of data were compared, and the percentage of area in which both sources of data consistently identified HIV hotspots was estimated.

## Results

### South Africa (local level)

**Fig. 1:**
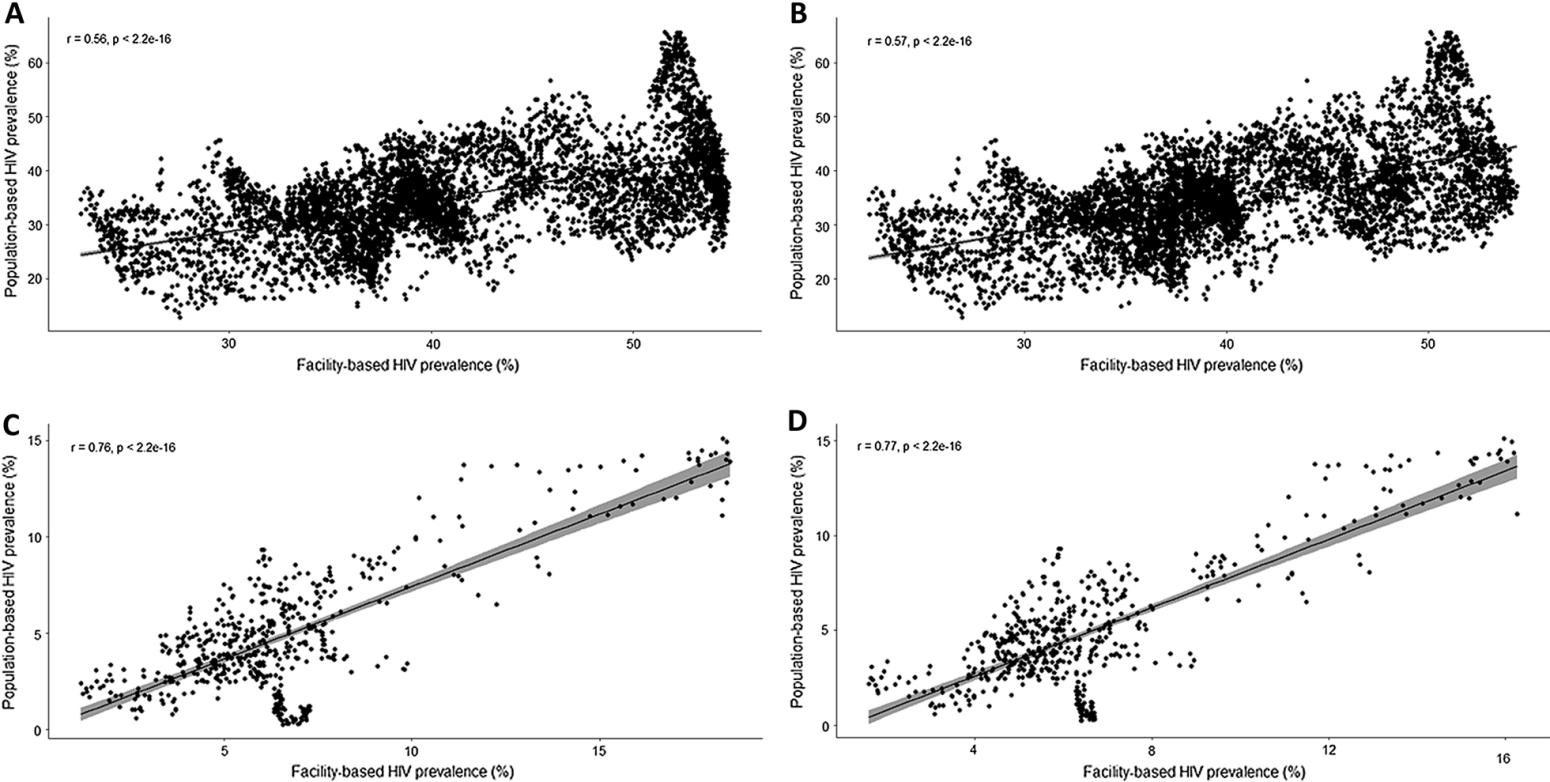
Comparisons between HIV prevalence estimations using population-based HIV prevalence data and clinic-based prevalence data. HIV prevalence estimations from the population-based data and clinic-based data derived from both models, kriging and cokriging, were extracted at each data point (black dots) included for the ACDIS study area and Tanzania. In **A** Africa Centre Demographic Information System study area using kriging, **B** Africa Centre Demographic Information System study area using cokriging, **C** Tanzania using kriging, **D** Tanzania using cokriging. The line in all figures indicates the fitted line between HIV prevalence estimations using population-based HIV prevalence data and clinic-based prevalence data

**Fig. 2:**
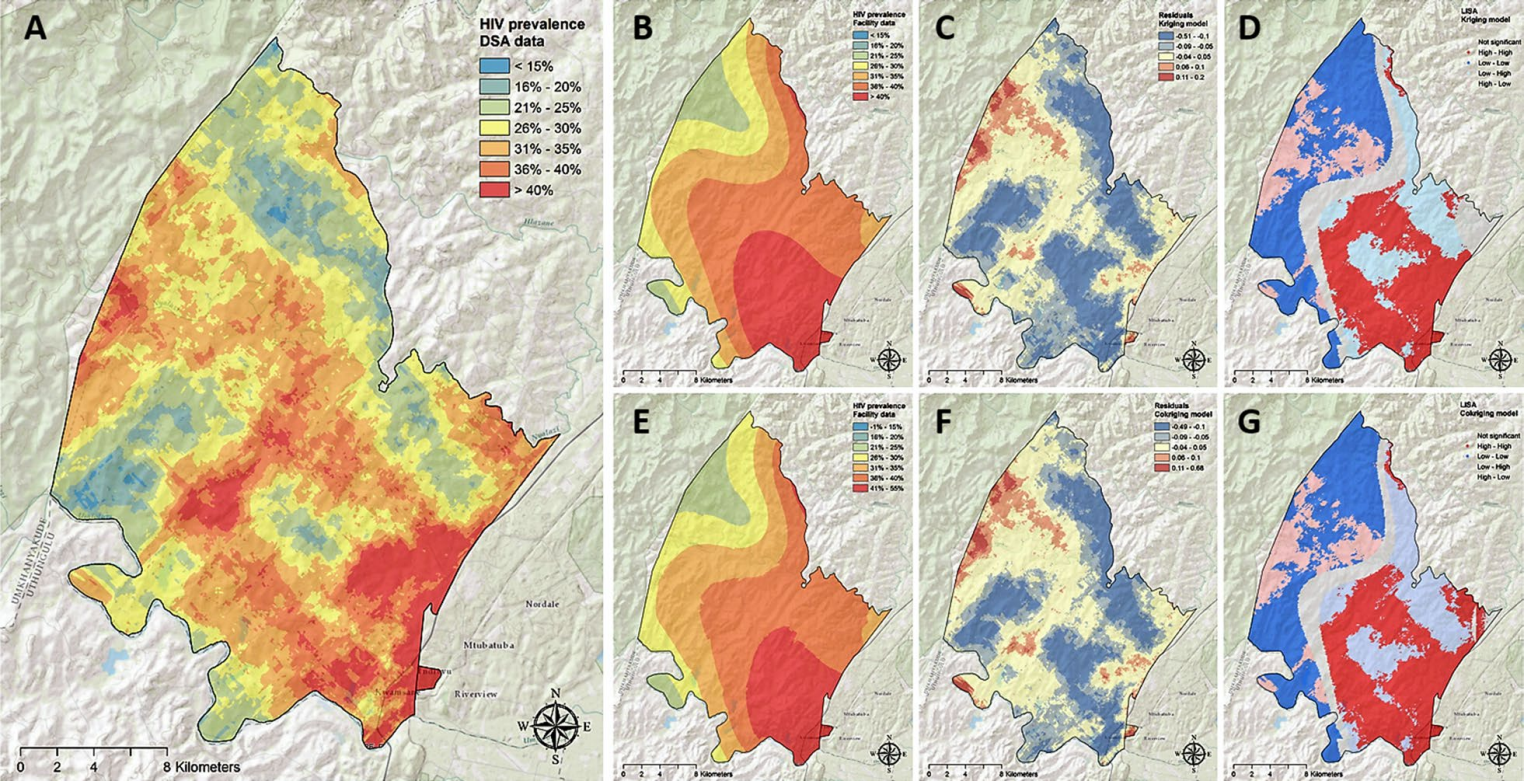
Continuous surface maps of **A** the estimated HIV prevalence using the Africa Centre Demographic Information System data, **B** kriging model for the estimated HIV prevalence using clinic-based data, **C** residuals from the kriging model, **D** LISA analysis of the kriging model, **E** cokriging model for the estimated HIV prevalence using clinic-based data, **F** residuals from the cokriging model, **G** LISA analysis of the cokriging model. Maps were created using ArcGIS^®^ software by Esri version 10.3 [22] (http://www.esri.com/)

Table 1 summarizes the estimated measures for map comparisons generated using both sources of data. Figure 1 illustrates the association between HIV prevalence estimated from population-based and clinic-based data for the ACDIS study area (A, B) and Tanzania (C, D). Continuous surface maps of HIV prevalence in the ACDIS study area are illustrated in Fig. 2. Semivariograms and histograms for pixel density distribution of HIV prevalence are included in Additional file (Additional file 1: Figures S5, S6 and S7). Mean pixel-level HIV prevalence in this area estimated using population-based data was 30.2% [95% confidence interval (CI) 16.5–43.9%]. Kriging interpolation maps revealed substantial geographical variation of the HIV epidemic in this small area of study (Fig. 2A), and identified areas with high HIV prevalence, particularly at the center, north-western, south-eastern parts of the study region.

**Table 1:** Summary of the estimated measures at local and national level estimated using kriging and cokriging methods

Measure	Local level (ACDIS study area)		National level (Tanzania)	
	Kriging	Cokriging	Kriging	Cokriging
PRCC^a^ (p value)	0.56 (p < 0.005)	0.57 (p < 0.005)	0.76 (p < 0.005)	0.77 (p < 0.005)
LISA consistency areas^b^	59%	61%	71%	77%
LISA inconsistency areas^c^	32%	30%	16%	10%
HIV ‘hotspot’ areas detected^d^	54%	56%	77%	84%

^a^Partial rank correlation coefficient (PRCC) comparisons between population-based data and clinic-based data estimations

^b^Percentage of area where the local indicator of spatial autocorrelation (LISA) estimations comparisons between population-based data and clinic-based data estimations were consistent with statistical significance

^c^Percentage of area where the local indicator of spatial autocorrelation (LISA) estimations comparisons between population-based data and clinic-based data estimations were inconsistent with statistical significance

^d^Percentage of areas with HIV prevalence in the upper quintile detected by the mode

Mean pixel-level HIV prevalence in this area estimated using clinic-based data was 35.6% (95% CI 21.9–49.3%). Kriging interpolation of clinic-based data identified the high burden areas of HIV infection located at the south-eastern part of the study area but failed to identify some other areas with high HIV prevalence, particularly at the north-western part of the study area (Fig. 2B). Residual analysis was consistent with this result and indicated that HIV prevalence interpolation using clinic-based data underestimated the HIV prevalence in the north-western part of the study area, but also overestimated the HIV prevalence in the high burden areas (Fig. 2C). LISA analysis indicated that estimations from kriging interpolation using these two types of data sources were consistent in identifying high or low burden areas in 59% of the study area (Fig. 2C). The estimations from these two models diverged in 32% of the study area: kriging interpolation using clinic-based data predicted high HIV burden areas in low HIV prevalence areas in 12% of the study area, and low HIV burden areas in high HIV prevalence areas in 20% of the study area (Additional file 1: Figure S6). There was a non-statistical significant spatial association in the remaining 9% of the study area. Inclusion of cofactors in the cokriging model moderately increased the accuracy of predictions (Fig. 2E, F), correctly identifying high or low burden areas in 61% of the study area (Fig. 2G, Additional file 1: Figure S6).

Map in Fig. 4A illustrates the location of the HIV ‘hotspots’ (areas with HIV prevalence in the upper quintile) in the ACDIS study area identified using population-based data. Kriging model map generated using clinic-based data (Fig. 4B) accurately predicted 54% of these high HIV burden areas, whereas cokriging model map (Fig. 4C) accurately located 56% of these high HIV prevalence areas.

### Tanzania (national level)

**Fig. 3:**
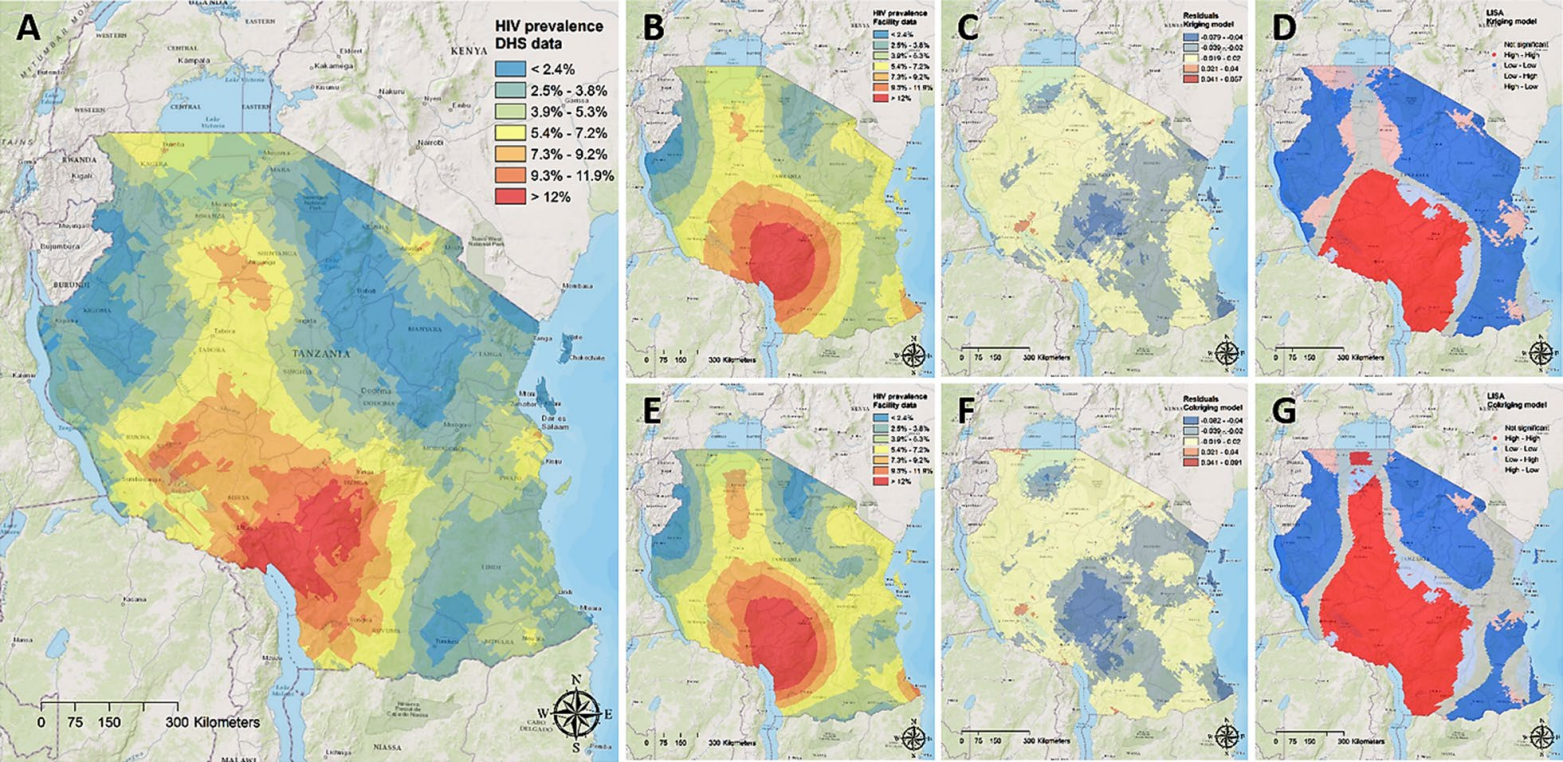
Continuous surface maps of **A** the estimated HIV prevalence using the Tanzania Demographic and Health Survey data, **B** kriging model for the estimated HIV prevalence using clinic-based data, **C** residuals from the kriging model, **D** LISA analysis of the kriging model, **E** cokriging model for the estimated HIV prevalence using clinic-based data, **F** residuals from the cokriging model, **G** LISA analysis of the cokriging model. Maps were created using ArcGIS^®^ software by Esri version 10.3 [22] (http://www.esri.com/)

Continuous surface maps for the HIV prevalence in Tanzania are illustrated in Fig. 3. Mean pixel-level HIV prevalence in this area estimated using population-based data was 5.2% (95% CI 1.0–9.4%). The burden of the infection appears to be concentrated in the south-western part of the country, between the districts of Mbeya and Iringa, where the HIV prevalence can reach more than 9% (Fig. 3A).

Mean pixel-level HIV prevalence in Tanzania estimated using clinic-based data was 6.5% (95% CI 1.6–11.3%). Kriging interpolation of clinic-based data identified all high burden areas of HIV infection detected using population-based data (Fig. 3B). However, residual analysis indicated that clinic-based data could overestimate the HIV prevalence in higher burden areas (Fig. 3C). LISA analysis indicated that estimations from kriging interpolation using DHS and clinic-based data were consistent identifying high or low burden areas in 71% of the area in Tanzania (Fig. 3D). The estimations from these two models diverged in 16% of the country area. Kriging interpolation using clinic-based data predicted high HIV burden areas in low HIV prevalence areas in 9% of the study area, and low HIV burden areas in high HIV prevalence areas in 7% of the study area (Additional file 1: Figure S7). There was a non-statistical significant spatial association in the remaining 13% of the study area. Similar to the results from the ACDIS study area, inclusion of cofactors in the cokriging model moderately increased the accuracy of predictions (Fig. 3E, F), consistently identifying high or low burden areas in 77% of the study area (Fig. 3G, Additional file 1: Figure S7).

**Fig. 4:**
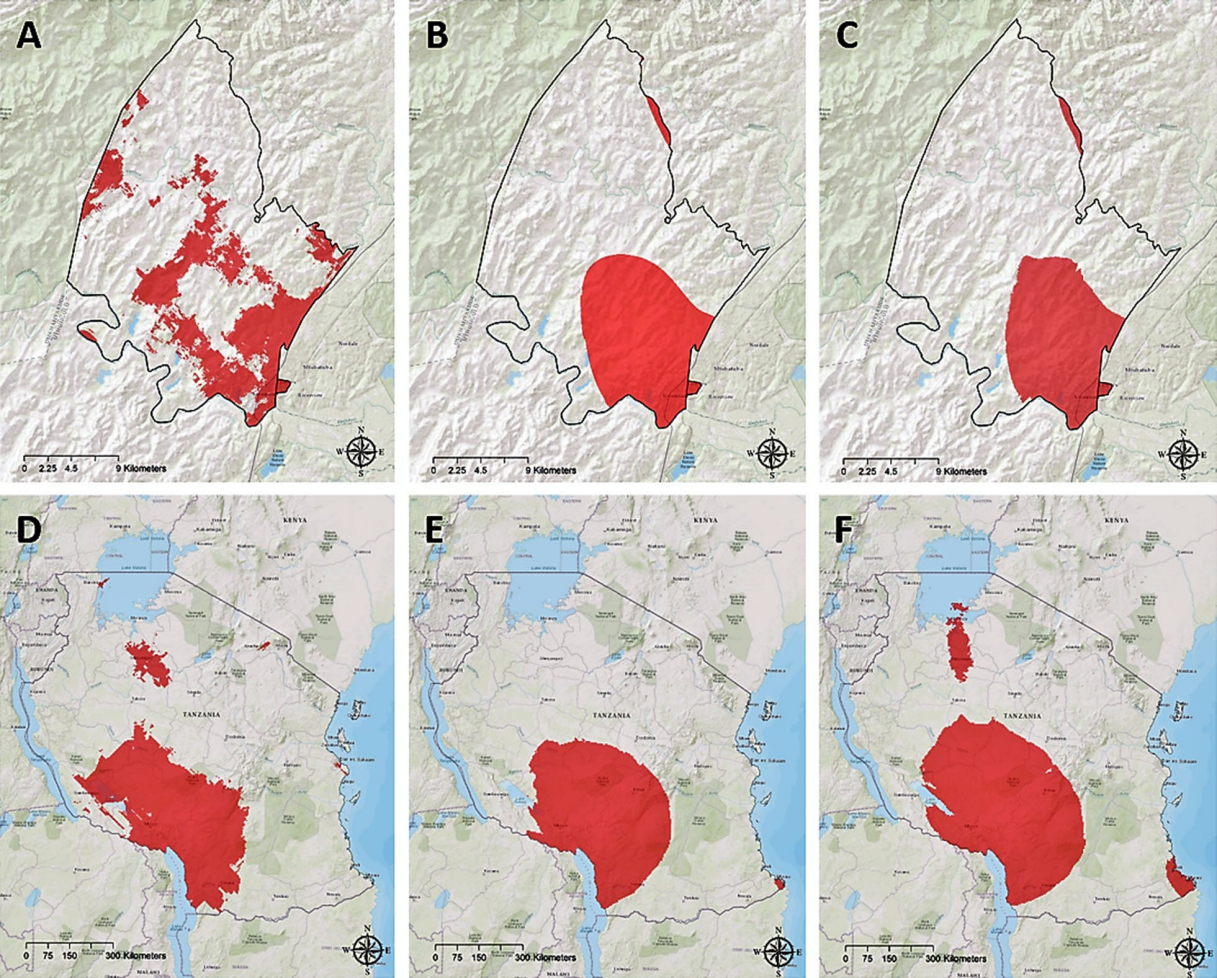
Areas with high HIV prevalence (≥ 80th percentile) in **A** kriging model for the estimated HIV prevalence in the Africa Centre Demographic Information System study area using population-based data, **B** kriging model for the estimated HIV prevalence in the Africa Centre Demographic Information System study area using clinic-based data, **C** cokriging model for the estimated HIV prevalence in the Africa Centre Demographic Information System study area using clinic-based data, **D** kriging model for the estimated HIV prevalence in Tanzania using population-based data, **E** kriging model for the estimated HIV prevalence in Tanzania using clinic-based data, **F** cokriging model for the estimated HIV prevalence in Tanzania using clinic-based data. Maps were created using ArcGIS^®^ software by Esri version 10.3 [22] (http://www.esri.com/)

Figure 4D maps the location of the HIV ‘hotspots’ (areas with HIV prevalence in the upper quintile) in Tanzania identified using population-based data. Kriging model map generated using clinic-based data (Fig. 4E) accurately predicted 77% of these high HIV burden areas, whereas cokriging model map (Fig. 4F) accurately located 84% of these high HIV prevalence areas.

## Discussion

Our results suggest that clinic-based data are able to capture the broad spatial structure of HIV epidemics in these hyperendemic settings. Analysis of this information accurately identified the high HIV burden areas (HIV ‘hotspots’), thereby offering a less expensive and readily available alternative source of data for the geographical identification of vulnerable populations at high risk of infection.

Accuracy of these predictions varied depending on the geographical scale in which the comparisons were conducted. Clinic-based HIV prevalence data successfully captured the broad spatial structure of the HIV epidemic in the areas studied, but the accuracy was reduced to some extent when the resolution of the geographical scale was increased (i.e. from national to local scale). For example, LISA results showed consistency of the HIV estimations in 59% of area in the ACDIS study area (local level), whereas it showed consistency of the HIV estimations in 71% of the area in Tanzania (national level). This discrepancy could be the result of reducing the number of healthcare facilities (data-points) in the analysis when the resolution of the scale is increased. For example, the HIV prevalence map generated for Tanzania included data from more than 100 healthcare facilities distributed across the country. In contrast, mapping the spatial distribution of HIV at sub-district level, focusing on a single town, only included 10 healthcare facilities. As a result, the statistical power and geographic resolution of the spatial interpolation could be reduced, amplifying discrepancies between population-based and clinic-based estimations. However, it is important to note that ACDIS has the unusual aspect of being bordered on the west by an uninhabited area, the Hluhluwe–Imfolozi national park. For that reason, there were no facility data points to improve boundary estimates on these areas. Despite this limitation, clinic-based data still captured most of the geographical variation of the HIV epidemic at local level, and located most of the high burden areas identified in previous studies, where the HIV epidemic is largely concentrated in the ACDIS study area [4, 5, 15, 16].

Inclusion of cofactors using cokriging method moderately increased the accuracy of spatial HIV prevalence predictions, particularly at national level. Nevertheless, it is important to note that this was an exploratory analysis, and only few cofactors were included in our study. Inclusion of more behavioral and biological cofactors associated with the risk of HIV infection such as male circumcision, condom use, life time number of sexual partners, and wealth index among others could effectively improve model predictions as it has been shown in previous studies [2, 9].

The primary limitation of the approach proposed here is the comparability across different sources of data collected from different sampled populations. Community-level population surveillances target the general population, whereas clinic-based surveillance systems collect data from specific subpopulations who seek care, such as pregnant women, or individuals at high risk of infection that are frequently tested or seeking treatment. Furthermore, while clinic-based data usually contain geographically representative of nearby populations, catchment areas are variable and HIV positive populations may be more likely to seek care at specific medical facilities with specialized services [28]. As expected, the clinic-based data over-estimated prevalence [29, 30]. Despite the fact that clinics are located following some decision rule, which is not completely spatially random regarding epidemic burdens, clinic-based data are still able to capture the spatial patterns, which is the ultimate goal of the approach proposed here. Lastly, we conducted our analysis using ESRI ArcGIS software, but alternative open access software such as QGIS (https://qgis.org/en/site/), GRASS (https://grass.osgeo.org/), and R (https://www.r-project.org/), among others, are suitable to conduct similar spatial analyses as the ones conducted in this study.

## Conclusions

Our results suggest that analysis of clinic-based data could provide robust estimations of the broad spatial structure of the HIV epidemic in hyperendemic settings. However, similar analyses should be conducted in other African settings to assess whether these patterns are observed elsewhere. Our study illustrates the potential utility of routine HIV testing data collected from different healthcare facilities to identify and monitor the high HIV burden areas. This methodology would overcome the challenging methodological and economic issues that accompany collecting community-level population-based data. Routine HIV testing data collected in different healthcare facilities as well as other sources of data from local small HIV sample surveys would allow for a rapid and cost-effective visualization of the epidemic, facilitating decision making to redefine resource allocation and surveillance systems, focused on the geographical HIV ‘hotspots’ that could be fueling the epidemic [5, 8, 12]. This approach may provide valid spatial prevalence estimates for geographical targeting where the burden of the infection is concentrated and where resources are needed the most.

## Additional file

Additional file 1. Supplementary materials.

## Abbreviations

HIV: human immunodeficiency virus
SSA: sub-Saharan Africa
UNAIDS: Joint United Nations Programme on HIV/AIDS
PEPFAR: United States President’s Emergency Plan for AIDS Relief
ACDIS: Africa Centre Demographic Information System
CAPRISA: AIDS Programme of Research in South Africa
DHIS: district health information system
DHS: Demographic and Health Survey
PRCC: partial rank correlation coefficient
LISA: local indicators of spatial autocorrelation
CI: confidence interval
