# Supplementary material for: Capturing the spatial variability of HIV epidemics in South Africa and Tanzania using routine healthcare facility data
Source: Int J Health Geogr. 2018 Jul 11;17:27. doi: 10.1186/s12942-018-0146-8 (PMC6042209; doi:10.1186/s12942-018-0146-8)
Supplement: Supplementary file 1 — Additional file 1. Supplementary materials. [file 12942_2018_146_MOESM1_ESM.pdf]

Supplementary Materials

Capturing the spatial variability of HIV epidemics in South Africa and Tanzania using routine healthcare facility data

Diego F. Cuadros, PhD[1, 2*], Ben Sartorius, PhD[3], Chris Hall, MSc[4], Adam Akullian, PhD[5], Frank Tanser, PhD[6, 7]

[1]*Deparment of Geography and Geographic Information Science, University of Cincinnati, Cincinnati, USA*

[2]*Health Geography and Disease Modeling Laboratory, University of Cincinnati, Cincinnati, USA*

[3]*Department of Public Health Medicine, School of Nursing and Public Health, University of Natal, Durban, South Africa.*

[4]*Geographical Information Systems & Science Program, Kingston University, London, UK*

[5]*Institute for Disease Modeling, 3150 139th Ave SE, Bellevue, USA*

[6]*School of Nursing and Public Health, University of KwaZulu-Natal, Durban, South Africa*

[7]*Africa Health Research Institute, University of KwaZulu-Natal, Durban, South Africa*

*To whom correspondence should be addressed. E-mail: diego.cuadros@uc.edu

Supplementary Methods

Local level data come from one of the most comprehensive demographic surveillance systems in Africa: the Africa Centre Demographic Information System (ACDIS) [1]. This surveillance system is located in Hlabisa subdistrict, one of the five subdistricts in the rural district of Umkhanyakude in northern KwaZulu-Natal, South Africa (Figure S1 A). The surveillance system has routinely collected socio-demographic, behavioural and epidemiological information on a population of about 90,000 participants within a circumscribed geographic area (438 km2) for over a decade (Figure S1 A). All participants included in the surveillance are geo-located to their respective homesteads of residence using the comprehensive Africa Centre geographic information system (accuracy <2m). Along with the ACDIS is a population-based HIV surveillance and sexual behavior survey which takes place annually. We included in our analysis the population-based HIV surveillance conducted in 2014. A total of 7,064 participants located in 5,174 homesteads were included in the sample. All individuals ≥15 years of age are eligible to be included in this survey. Of those participants contacted, 60% agree to be tested at least once. After obtaining written informed consent, blood by finger prick is collected by field workers and prepare dried blood spots for HIV testing according to the UNAIDS and WHO Guidelines for Using HIV Testing Technologies in Surveillance. The data source for the healthcare facility data for this study area was district health information system (DHIS). DHIS gathers aggregate monthly data from each facility in each district across the country. It includes routine data elements for HIV testing and HIV test results in both general population as well as among pregnant women. HIV testing in healthcare facilities in South Africa is conducted using a rapid HIV test, in which a serial testing algorithm approach is followed.

Country level data come from the Demographic and Health Survey conducted in Tanzania (TDHS) in 2011-12 [2]. For this sampling, subjects were enrolled via a two-stage sampling procedure to select a total of 568 households. The global positioning system was used to identify and record the geographical coordinates of each DHS sample location (Figure 1). Men and women in the selected households were eligible for the study. The study population used in this study consisted of 17,745 individuals in Tanzania (9,756 women and 7,989 men). Anonymous HIV testing was performed with the informed consent of all sampled individuals. HIV serostatus was determined by testing with the enzyme-linked immunosorbent assay (ELISA) Vironostika Uniform 2 Ag/AB. All samples that tested positive and a random sample of 10% of samples that tested negative were retested with a second ELISA, the Enzygnost® HIV Integral II assay (Siemens). Positive samples on both tests were classified as HIV positive. If the first and second tests were discordant, the two ELISAs were repeated; if the results remained discordant, a confirmatory test, the HIV 2.2 western blot (DiaSorin), was administered. Further details related to the DHS methodology, study design, and data can be found elsewhere [2-4]. Healthcare facility data from Tanzania come from antenatal clinic (ANC) surveillance where HIV testing was routinely conducted to pregnant women attending these clinics in 2010. After obtaining an informed verbal consent from the pregnant woman at the clinic, about 3–5 ml of blood was collected and sent to the National Health Laboratory Quality Assurance and Training Centre NHL-QACTC). This Laboratory works hand in hand with the National AIDS Control Programme (NACP) to ensure high quality HIV testing services. Protocols for samples collections and testing are described elsewhere [5].

Supplementary figures

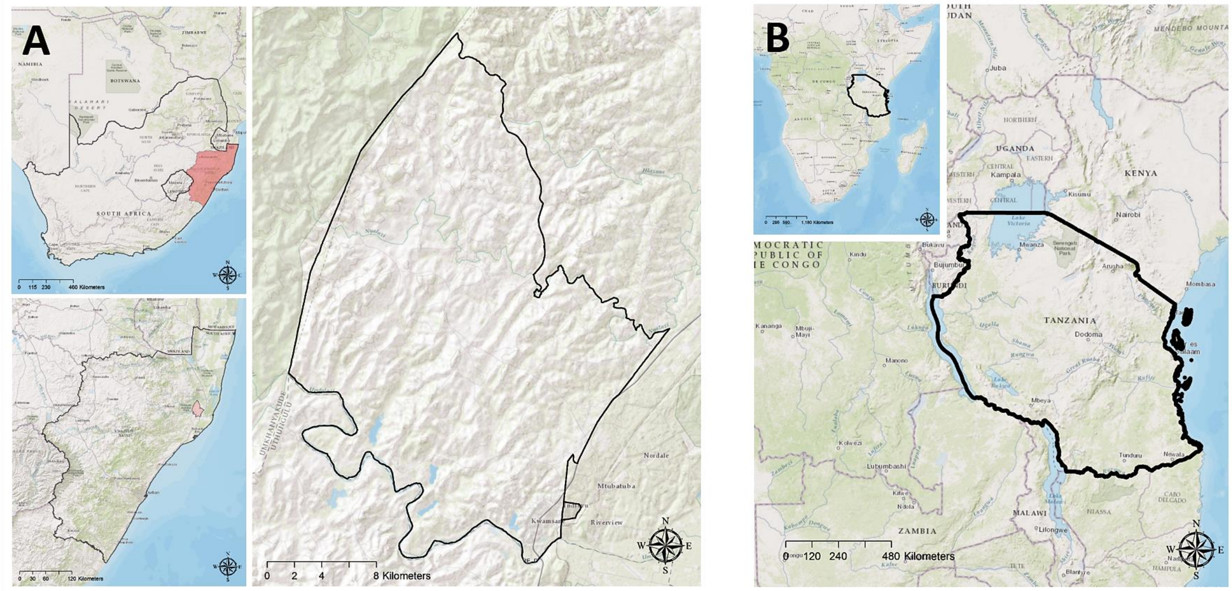

Figure S1. Study area for A) The Africa Centre Demographic Information System, Kwazulu-Natal, South Africa, and B) Tanzania. Maps were created using ArcGIS® software by Esri version 10.3 [6] (http://www.esri.com/).

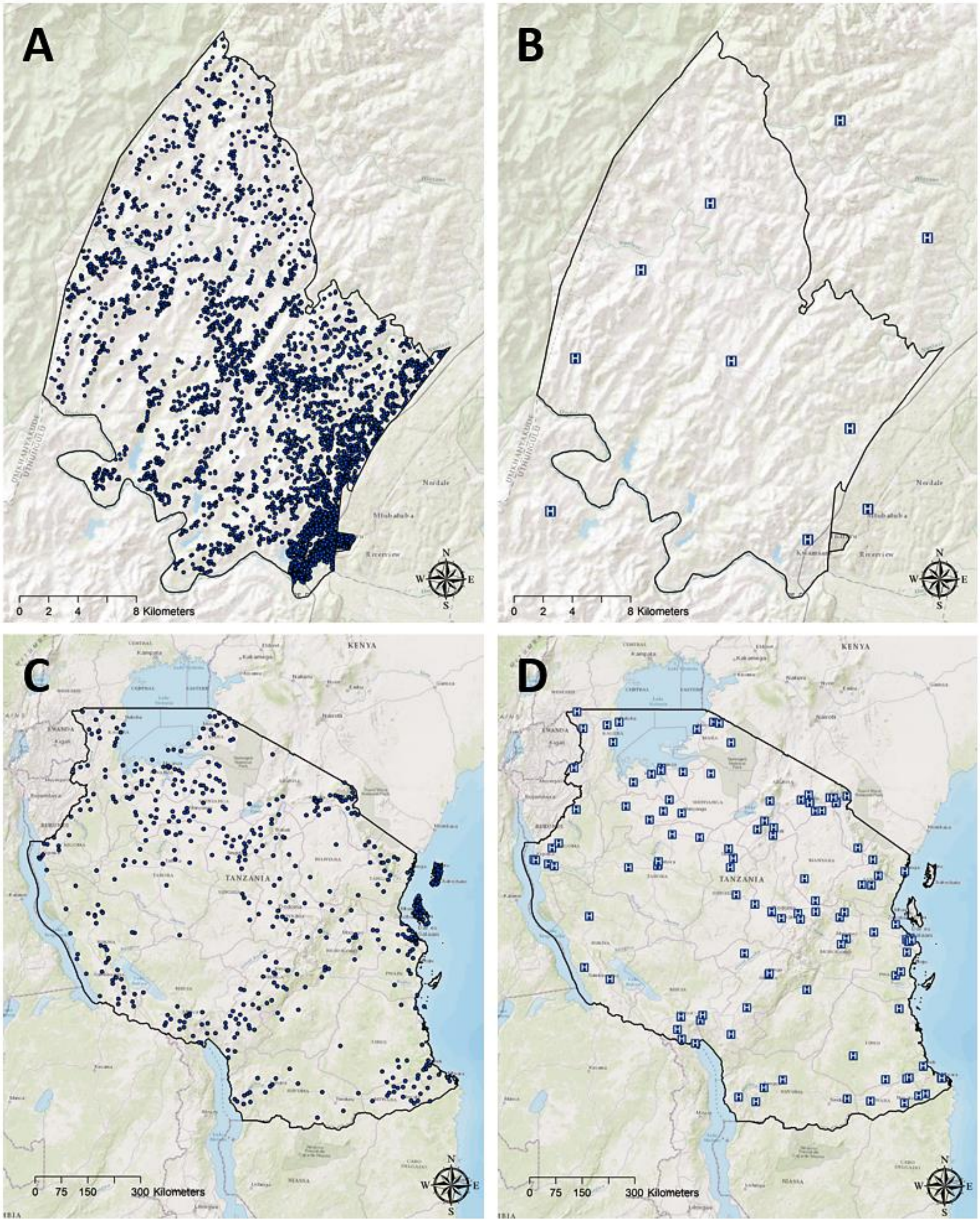

Figure S2. Sampling locations. A) Geographical distribution of the homesteads of the participants included in the study, and B) location of the healthcare facilities in a rural community in South Africa. C) Geographical distribution of the Demographic and Health Survey sample data points conducted in Tanzania, and D) location of healthcare facilities in Tanzania. Maps were created using ArcGIS® software by Esri version 10.3 [6] (http://www.esri.com/)

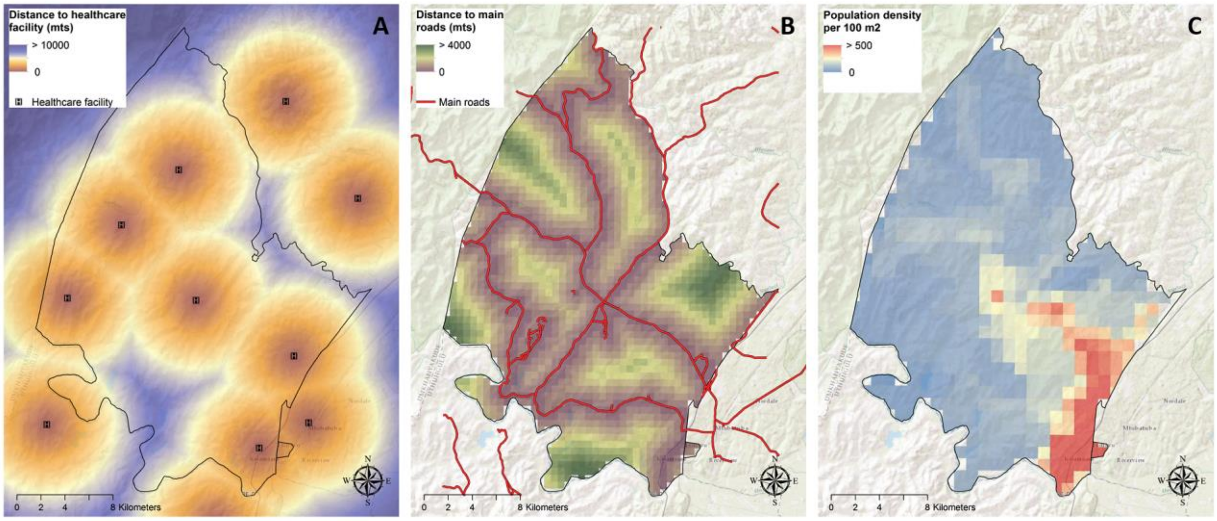

Figure S3. Continuous surface maps for the cofactors included in the cokriging model for predicting the HIV prevalence distribution in a rural community in South Africa. A) distance to the closes healthcare facility, B) distance to main roads, and C) population density. Maps were created using ArcGIS® software by Esri version 10.3 [6] (http://www.esri.com/)

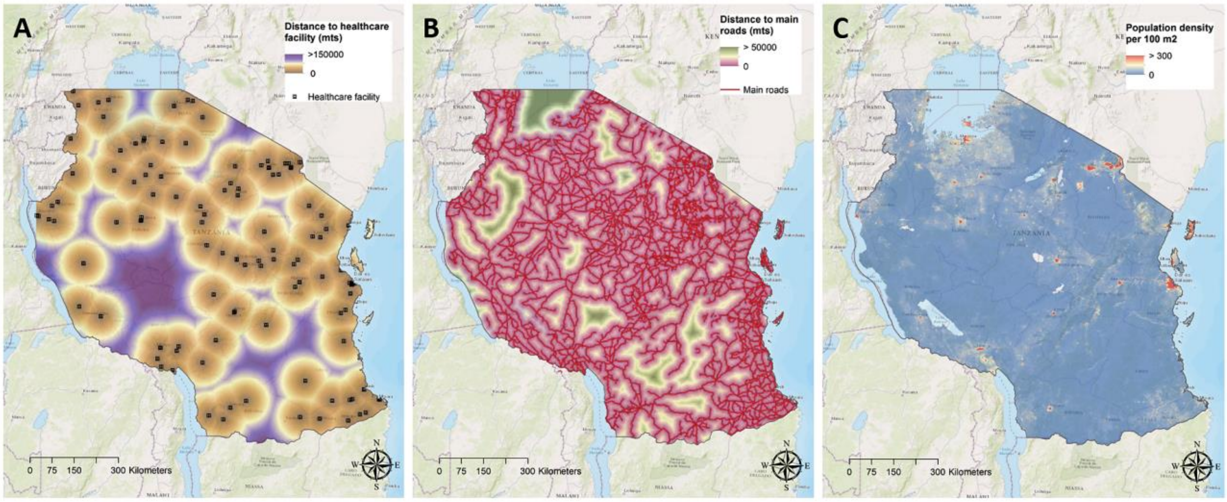

Figure S4. Continuous surface maps for the cofactors included in the cokriging model for predicting the HIV prevalence distribution in Tanzania. A) distance to the closes healthcare facility, B) distance to main roads, and C) population density. Maps were created using ArcGIS® software by Esri version 10.3 [6] (http://www.esri.com/)

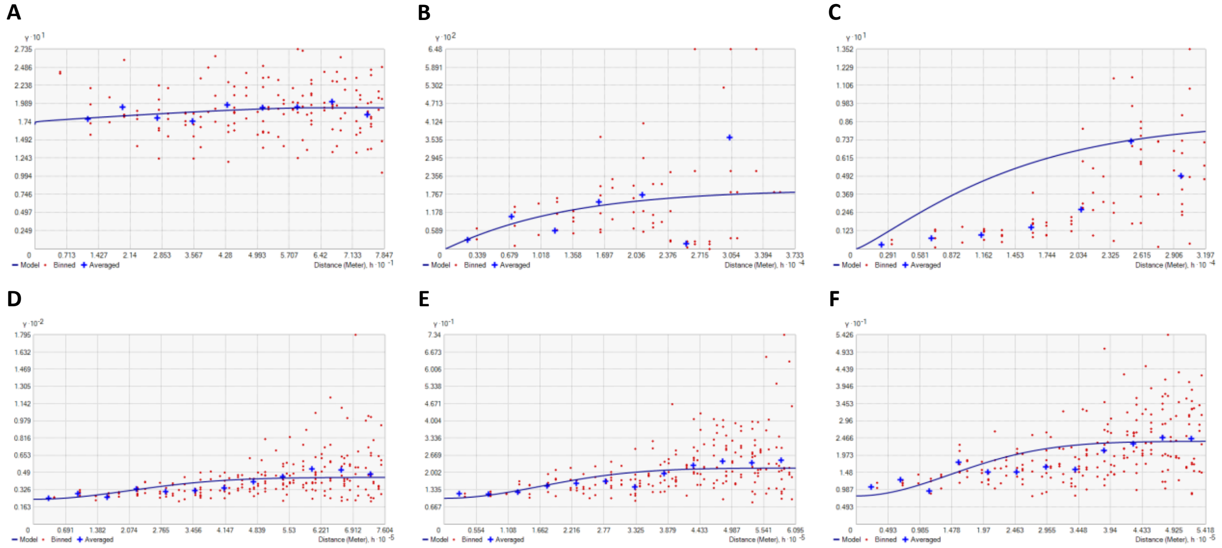

Figure S5. Semivariograms using data from A) the Africa Centre Demographic Information System, B) healthcare facilities enclosed in the study area of the Africa Centre Demographic Information System, C) healthcare facilities enclosed in the study area of the Africa Centre Demographic Information System including cofactors (cokriging model), D) the Tanzania Demographic and Health Survey, E) healthcare facilities in Tanzania, and healthcare facilities in Tanzania including cofactors (cokriging model)

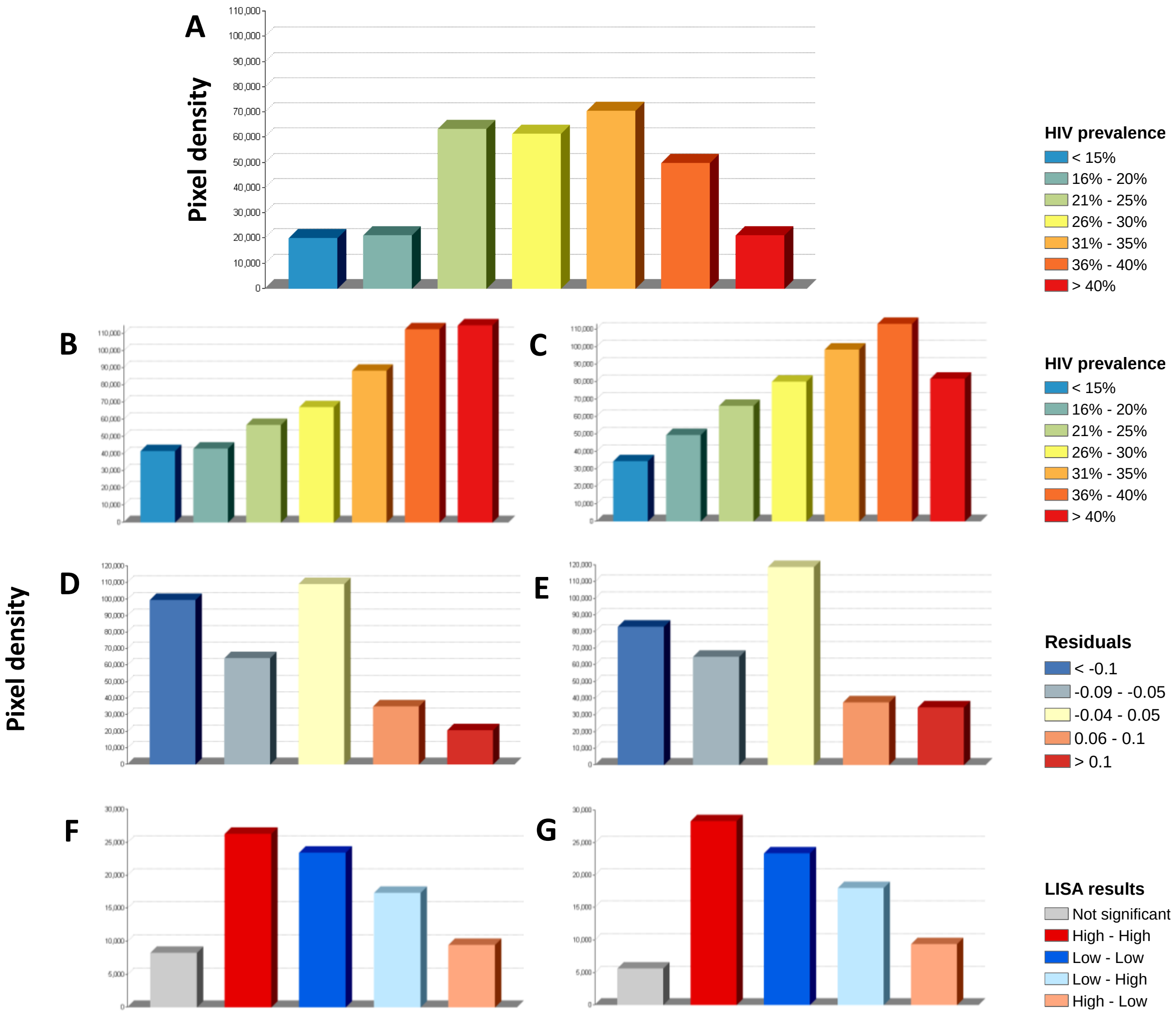

Figure S6. Pixel density distribution for A) the estimated HIV prevalence using the Africa Centre Demographic Information System data, B) kriging model for the estimated HIV prevalence using clinic-based data, C) cokriging model for the estimated HIV prevalence using clinic-based data, D) residuals from the kriging model, E) residuals from the cokriging model, F) LISA analysis of the kriging model, and G) LISA analysis of the cokriging model

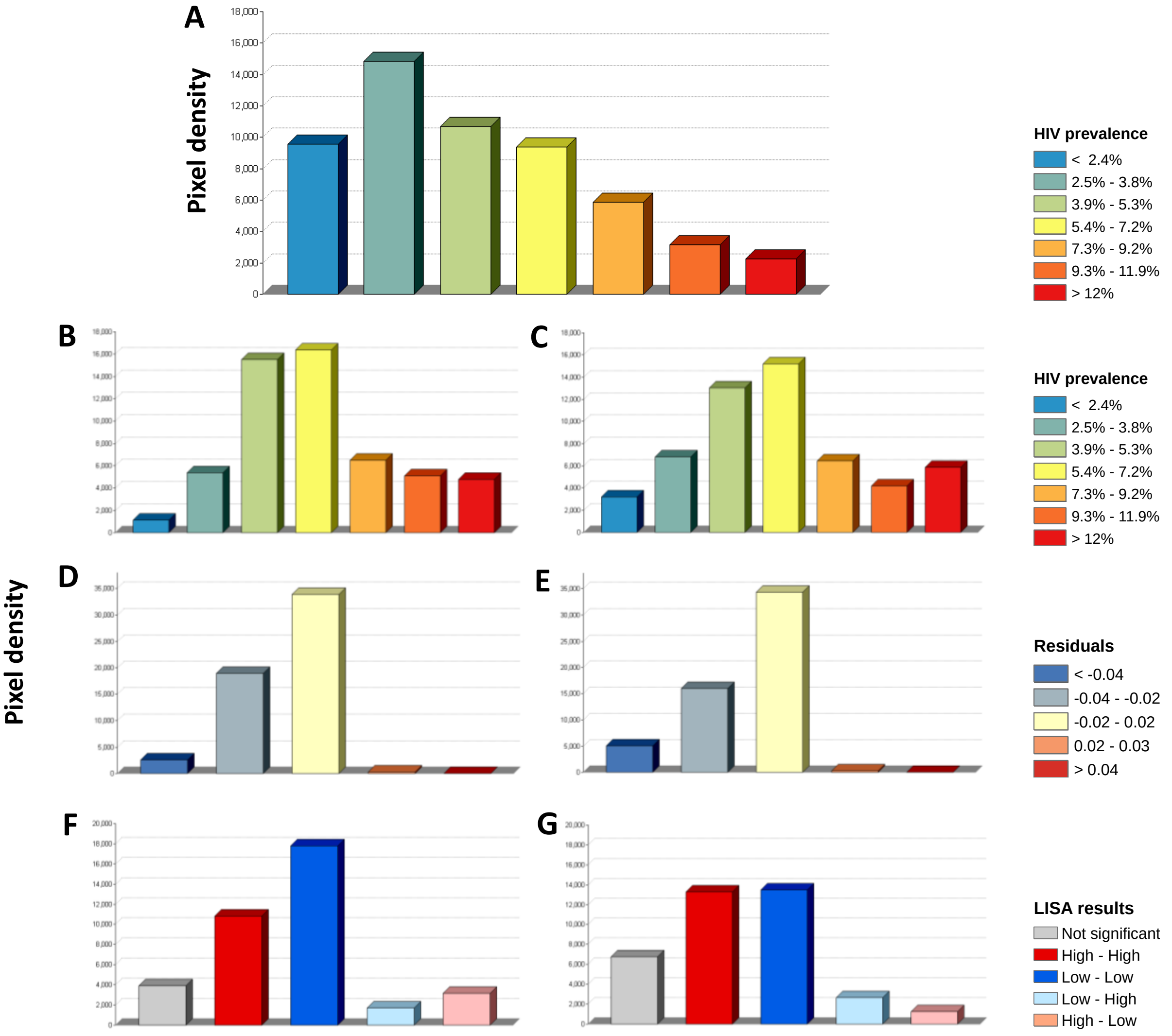

Figure S7. Pixel density distribution for A) the estimated HIV prevalence using the Tanzania Demographic and Health Survey data, B) kriging model for the estimated HIV prevalence using clinic-based data, C) cokriging model for the estimated HIV prevalence using clinic-based data, D) residuals from the kriging model, E) residuals from the cokriging model, F) LISA analysis of the kriging model, and G) LISA analysis of the cokriging model

References

1. Aral, S.O., E. Torrone, and K. Bernstein, *Geographical targeting to improve progression through the sexually transmitted infection/HIV treatment continua in different populations.* Current Opinion in HIV and AIDS, 2015. **10**(6): p. 477-482.
2. Tanzania Commission for AIDS, Z.A.C., National Bureau of Statistics, Office of Chief Goverment Statistician, ICF international, *Tanzania HIV/AIDS and Malaria Indicator Survey 2011-12*. 2013, Calverton, MD: ICF International.
3. Tanzania Commission for AIDS, Z.A.C., National Bureau of Statistics, Office of Chief Goverment Statistician, Macro International Inc., *Tanzania HIV/AIDS and Malaria Indicator Survey 2007-08*. 2008, Calverton, MD: Macro International Inc.
4. Tanzania Commission for AIDS, N.B.o.S., ORC Macro, *Tanzania HIV/AIDS indicator Survey 2003-04*. 2005, Calverton, MD: ORC Macro.
5. *The United Republic of Tanzania, Ministry of Healt. National guidelines for voluntary counseling and testing, 2005.* National AIDS Control Porgramme (NACP), 2005.
6. ESRI, *ArcGIS 10.x. Redlands, CA, USA: ESRI.* 2004.
